# Clinical study for external washing by traditional Chinese medicine in the treatment of multiple infectious wounds of diabetic foot: Study protocol clinical trial (SPIRIT compliant)

**DOI:** 10.1097/MD.0000000000019841

**Published:** 2020-04-24

**Authors:** Yuan Zhang, Haipo Yuan, Jian Kang, Hongyan Xie, Xinhua Long, Luguang Qi, Chunguang Xie, Guangming Gong

**Affiliations:** a Hospital of Chengdu University of Traditional Chinese Medicine, Chengdu; b Neijiang Hospital of Traditional Chinese Medicine, Neijiang, Sichuan Province, P.R. China.

**Keywords:** Chinese herbal medicine, diabetic foot, diabetic foot infection, foot bath decoction, randomized controlled trial

## Abstract

**Background:**

Diabetic foot (DF) is among the most serious complications of type 2 diabetes. DF infection (DFI) is a key factor in the deterioration and development of DF, so controlling infection plays an important role in the treatment of the disease. Traditional Chinese medicine foot bath has been widely used in China as a complementary and alternative therapy to improve circulation and infection control of DF. However, the existing evidence shows that its efficacy and safety are still insufficient. We report a study protocol about a multicenter, double-blind, randomized, placebo controlled trial which aims to make well-designed clinical trials to evaluate the efficacy and safety of herbal medicine foot bath decoction (FBD) and explore the mechanism of external washing of Chinese herbs in DFI.

**Methods:**

This study is a multicenter, double-blind, randomized, placebo controlled clinical trial in which 60 eligible participants were randomly divided into an experimental group and control group at a 1:2 ratio. Both groups received the same basic treatment for DF disease, the experimental group used FBD and ordinary dressing changes, while half of the patients in the control group received placebo and ordinary dressings, and the other half received placebo and silver ion dressings. Patients in both groups will be evaluated weekly for efficacy during the intervention. The primary efficacy indicators include the types of wound pathogens, interleukin 6 and tumor necrosis factor α. Secondary efficacy indicators included blood glucose, blood lipids, wound area, lower extremity blood vessel diameter, blood flow speed, walking speed, walking distance, and traditional Chinese medicine syndrome scores. We will also conduct a safety evaluation of the drug at the end of the trial.

**Discussion:**

This multicenter, double-blind, randomized, placebo clinical trial not only provides data on the efficacy and safety of FBD, but also provides a novel treatment strategy for clinicians and DF patients.

## 1 Introduction

Diabetic foot (DF) is a globally prevalent chronic complication that causes an increased risk of lower limb amputation and death in diabetic individuals.^[1]^ It is reported that about 1 in 4 people with diabetes will suffer from DF during their lifetime, and the point prevalence rate is almost 2%.^[2,3]^ Along with this, engaging in medical care related to DF has placed a huge economic burden to patients and the society.^[4]^ DF usually has a poor prognosis. According to a retrospective study in China, the proportion of amputations caused by DF is 19.03% to 27.3%.^[5,6]^ DF infection (DFI) is the foremost cause of the occurrence and progression of DF ulcers, and the susceptibility factors are closely related to autoimmune deficiency, neuropathy, and arterial disease.^[7]^

The pathologic process of DF is very complicated, involved in damaged skin, muscle, tendon, bone, nerve, and blood vessel. The DFI refers to the invasion of microorganisms and its reproduction in host tissues, which usually begins with the dehiscence of the shielding skin envelope and plays an indispensable role in the above process.^[8]^ The treatment of DF advocates a multidisciplinary combination of endocrinology, dermatology, vascular surgery, and orthopedics,^[9–11]^ which involves controlling blood glucose, reducing blood pressure, ulcer debridement, reducing stress, controlling infection, and rebuilding blood circulation.^[12,13]^Although the use of antibiotics is an indispensable means of controlling DFI, the abuse of antibiotics and the presence of drug-resistant bacteria make this treatment process more difficult. In addition, given that some antibiotics may cause liver damage, nephrotoxicity, and a range of adverse reactions of the digestive system in the digestive tract.^[14]^ Therefore, seeking personalized therapy and new complementary drugs to control infection have not stopped.

In recent years, most patients with DF in China are adopting a treatment method that combines traditional Chinese medicine (TCM) and western medicine. Foot bath, with a history of thousands of years, is widely used in traditional medicine to treat surgical diseases, especially infectious wounds. Foot bath decoction (FBD) is a TCM compound consisting of Raw Rhubarb (Shengdahuang), Coptidis Rhizoma (Huanglian), Fructus Forsythia (Lianqiao), aluminum potassium sulfate (Kufan), Pseudobulbus Cremastrae Seu Pleiones (Shancigu). Modern pharmacologic researches have shown that Rhein, the main active ingredient of Raw Rhubarb, has obvious effects in promoting metabolism, anti-inflammatory, antibacterial, analgesic, and improving circulation.^[15,16]^ Coptidis Rhizoma has been used to treat various inflammatory disorders and related diseases for a thousand years, and various modern studies have demonstrated that Coptidis Rhizoma has wide pharmacologic activities, including antibacterial, antifungal, antiatherosclerosis, antimyocardial ischemia/reperfusion injury, antidiabetic, anti-inflammation, antioxidation.^[17,18]^ Considering that Fructus Forsythia has a strong anti-inflammatory effect and acts as a broad spectrum antimicrobial agent, it is widely used in chronic and acute inflammation. Some research confirm that Fructus Forsythia's anti-inflammatory activity ranks among top 5 of 81 tested Chinese medicines.^[19,20]^ Numerous animal experiments present that aluminum potassium sulfate can inhibit the growth of anaerobic and facultative anaerobic bacteria.^[21]^ A variety of active compounds in Pseudobulbus Cremastrae Seu Pleiones have antibacterial effects, especially against *Pseudomonas aeruginosa*, *Staphylococcus epidermidis*, and *Staphylococcus aureus*.^[22]^ Therefore, the FBD has the functions of clearing heat, detoxifying, and eliminating evil from the theory of TCM. It is reasonable to assume that FBD is effective in the treatment of DFIs. However, there is no direct evidence to support its efficacy and safety.

Recently meta-analysis has shown that TCM foot bath can significantly improve the sensory nerve conduction velocity, motor nerve conduction velocity, and neuropathy syndrome scores in patients with diabetic peripheral neuropathy, and has certain advantages in alleviating its clinical symptoms.^[23]^ Foot bath can inhibit bacteria through the environmental changes on the surface of foot ulcers. Several studies have shown that the anti-inflammatory effect of herbal medicine foot bath may be achieved by lowering blood glucose and reducing the number of hypersensitive C-reactive protein and white blood cells.^[24]^ Based on the above conclusions, we hypothesized that FBD can both regulate and inhibit local bacteria to promote healing of DF wounds.

## 2 Methods and design

### 2.1 Design

**Figure 1:**
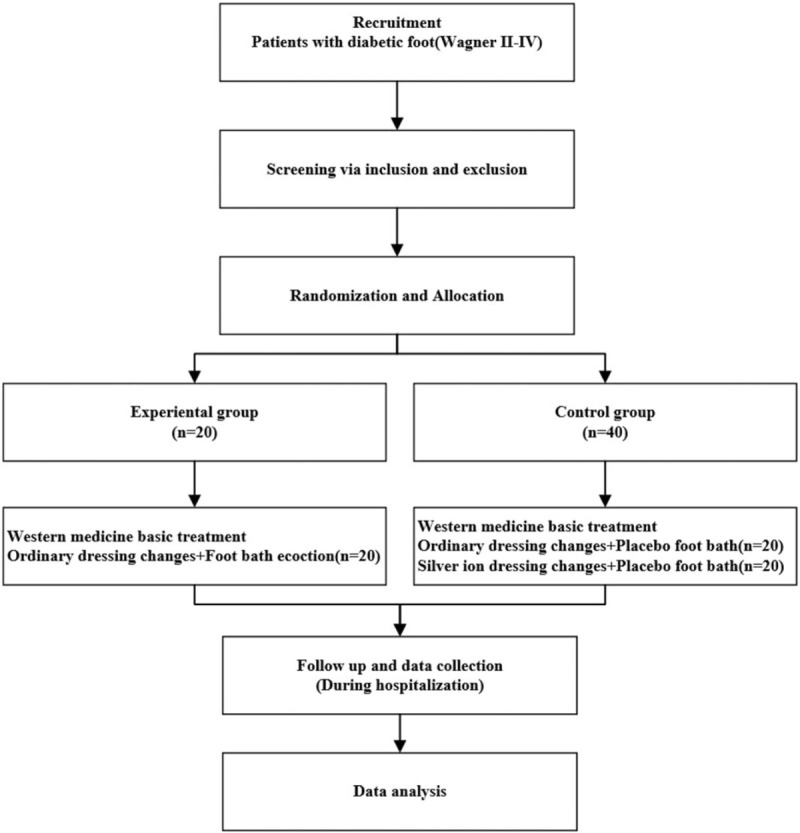
Flow chart of the study.

This study is a multicenter, double-blind, randomized, placebo controlled clinical trial, and the protocol was registered in Chinese Clinical Trial Registry on July 16, 2019, ChiCTR1900024557. Sixty eligible participants signed the informed consent and were randomly assigned to the experimental or control group at a ratio of 1:2. Both groups received the same basic treatment for DF disease, the experimental group was additionally washed with FBD and ordinary dressing changes, while half of the patients in the control group received placebo and ordinary dressings, and the other half received placebo and silver ion dressings. The purpose of this trial is to explain the efficacy and safety of herbal medicine FBD for DFI compared with western medicine (WM) alone. This study will adhere to the standard protocol items: Recommendations for Interventional Trials (SPIRIT) 2013 statement, and flow chart of the study will be used to show the screening process (Fig. 1).

### 2.2 Ethics and dissemination

The study is in compliance with the Declaration of Helsinki (Edinburgh 2000 version). The program was approved by the Ethics Committee of Sichuan Traditional Chinese Medicine Regional Ethics Review Committee Chengdu University of TCM Teaching Hospital Ethics Committee (Code 2019KL-020). If there are any additions or amendments to the protocol during the research, the consent of the ethics committee must be obtained again.

### 2.3 Recruitment

This study is a multicenter clinical trial. The recruitment area involves 3 hospitals, namely Hospital of Chengdu University of Traditional Chinese Medicine, Shuangliu District Traditional Chinese Medicine Hospital and Mianyang Traditional Chinese Medicine Hospital. It should be noted that the main sponsor is Hospital of Chengdu University of Traditional Chinese Medicine. Recruit participants by posting posters at various hospitals. Before signing an informed written consent, participants will be familiar with all the details of the clinical study and the potential risks and benefits.

### 2.4 Sample size

This study is a superiority clinical trial with interleukin 6 (IL-6) as the main efficacy indicator, so we chose the mean superiority test of 2 independent samples to estimate the sample size. According to the previous relevant references,^[25]^ the optimal response rate was set to 0.04. The mean values of the experimental group and the control group were 6.14 and 6.21, and the combined standard deviation of the 2 groups was 0.14. At the same time, set *a* to 0.025 and *b* to 0.2. Enter the above values into the Power Analysis and Sample Size version 11.0 (PASS 11.0) software to calculate the sample size for the experimental group as 20 and the control group as 40. Finally, the total number of study samples is determined to be 60 cases.

### 2.5 Randomization

The random sequence will be generated by PROCPLAN in the SAS software and the entire process is operated by the teacher of the Statistics Department of Chengdu University of Traditional Chinese Medicine. The results of the random sequence will be hidden and spread using opaque envelopes.

### 2.6 Blinding

The study was a double-blind clinical trial. All researchers, subjects, and statisticians were blinded throughout the intervention period. Unless participants have serious adverse events to receive emergency treatment, they cannot eliminate blindness. Both the FBD and the placebo granules will be produced by the Pharmacy Department of Hospital of Chengdu University of Traditional Chinese Medicine. Both are ensured to be consistent in appearance, shape, or odor and specifications. Moreover, ordinary dressings must also be no different from silver ion dressings in terms of appearance and smell. It is worth mentioning that research members cannot discuss with the participants their possible treatment options.

### 2.7 Diagnostic criteria

#### 2.7.1 Western medicine diagnostic criteria

Participants not only need to comply with the diagnostic criteria for diabetes WHO diagnostic criteria in 1999^[26]^ (Table 1), but also follow the diagnostic and grading criteria for DF produced by The International Working Group on the Diabetic Foot (IWGDF)^[27]^ (Table 2).

**Table 1:**
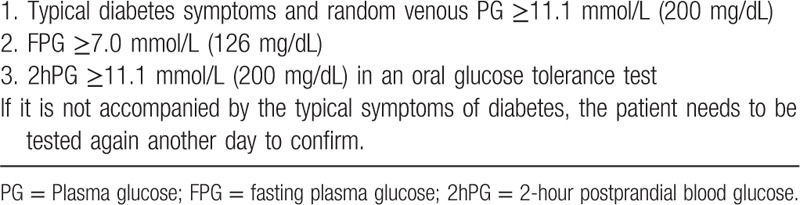
WHO diagnostic criteria for diabetes.

**Table 2:**
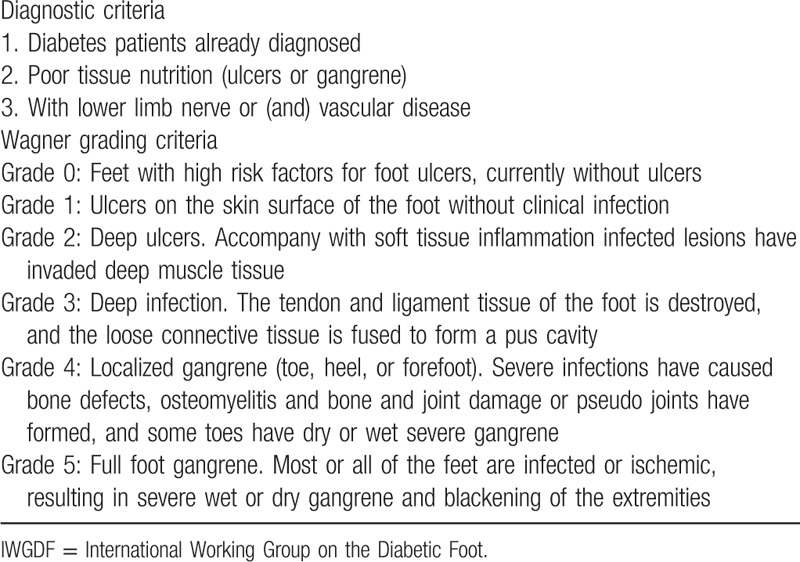
IWGDF diagnostic and grading criteria for diabetic foot.

#### 2.7.2 TCM diagnostic criteria

Referring to classification criteria for gangrene in the “Guiding principles for new drug clinical research of TCM,”^[28]^ the detailed diagnostic items are shown in Table 3.

**Table 3:**
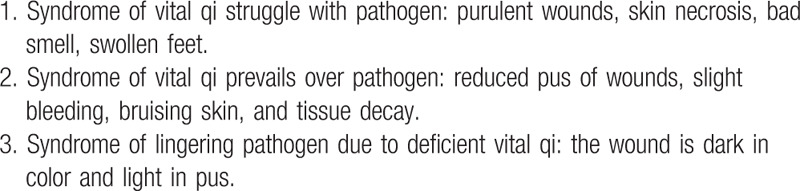
Classification criteria of gangrene for traditional Chinese medicine.

### 2.8 Eligibility criteria

Inclusion criteria:

1. Diagnosis of DF according to WM and TCM.
2. According to Wagner classification of DF, patients with stages II, III, IV are eligible.
3. Aged between 18 and 75 years old.
4. Voluntarily participate in the test and sign the informed consent.

Exclusion criteria:

1. Patients with cardiac and renal insufficiency, acute coronary syndrome, cerebrovascular accident, hematopoietic system disorders and mental diseases, malignant tumors and end-stage patients.
2. Women who are breastfeeding, pregnant, or preparing for pregnancy.
3. Allergies for known drugs or experimental drugs.
4. Uncooperative during the experiment.
5. Having a history of alcoholism or drug abuse.
6. Having a history of another clinical study in the previous 1 month.

### 2.9 Termination and withdrawal criteria

All participants were informed that they had the right to drop out of the study before starting the experiment. The test will also be withdrawn or terminated if the following criteria occur.

1. Side effects and adverse reactions occur, which are difficult for patients to tolerate.
2. Serious adverse events occurred.
3. Complicated with other diseases during the test.
4. Patients with poor compliance and failed to perform research protocol as required.

### 2.10 Test drugs

Test drugs including FBD and placebo were prepared by the Department of Pharmacy of the Affiliated Hospital of Chengdu University of Traditional Chinese Medicine. The composition of the FBD is as follows: Raw Rhubarb (Sheng dahuang) 20 g, Coptidis Rhizoma (Huang lian) 15 g, Fructus Forsythia (Lian qiao) 15 g, Aluminum potassium sulfate (Ku fan) 10 g, Pseudobulbus Cremastrae Seu Pleiones (Shan cigu) 10 g. All herbs are mixed, soaked, cooked, and made into a single-dose pouch of uniform specifications by the pharmacy department, each bag is about 150 mL. Placebos are made from starch without active ingredients and added a variety of food colors. The placebo was consistent with the FBD in color, odor, and dosage. Furthermore, the ordinary dressings and silver ion dressings should also have no difference in appearance and smell.

## 3 Interventions

### 3.1 Treatment plan

Both groups received based on dietary treatments recommended by the Diabetes UK evidence-based nutrition guidelines for the prevention and management of diabetes,^[29]^ and controlled blood glucose, lowered blood lipids, adjusted blood pressure, and suppressed infections according to individual conditions during the trial period. At the same time, DF ulcers can be treated by surgical debridement to remove necrotic tissue, improve lower limb circulation and a routine dressing every 2 to 3 days according to the Diabetic Foot Ulcer Treatment Guidelines.^[30]^

Experimental group: The participants in experimental group took a foot bath 1 to 2 times a day. Each bag of FBD was dissolved in 300 mL of warm water for 30 minutes, then cover the wound with a ordinary dressing.

Control group: The patients in the control group will be given a placebo decoction for foot bath twice daily for 30 minutes. The immersion method is the same as the experimental group. In contrast, half of the patients covered the wound with an ordinary dressing and half with a silver ion dressing.

### 3.2 Outcome measures

Primary efficacy outcomes:

1. IL-6 and tumor necrosis factor α (TNF-α) were detected by enzyme-linked immunosorbent assay and were measured before and each week after treatment during entire treatment.
2. Wound pathogen distribution: Collect samples of ulcers and use a fully automatic microbiologic tester for bacterial detection once a week.

Secondary efficacy outcomes:

1. Blood glucose and blood lipid: The blood glucose, total cholesterol, triglyceride, low-density lipoprotein cholesterol, and high-density lipoprotein cholesterol are detected by a fully automatic biochemical analyzer.
2. Wound area: Long multiplied by high area. First take a picture of the wound and then read the data with Image J software. The above operations are performed by the same doctor.
3. Lower extremity blood vessel diameter, blood flow velocity: detected by lower extremity blood vessel Doppler ultrasound.
4. Walking speed, walking distance: Make patients walk on a flat ground with a scale, and calculate the distance and speed.
5. TCM syndrome score: Recorded with TCM Syndrome Score Evaluation Form.

### 3.3 Safety assessment

Safety indicators will be reflected by blood cell analysis, urine routine, stool routine, liver function indicators, and kidney function indicators.

### 3.4 Endpoint outcomes

Endpoint events refer to adverse cardiovascular and cerebrovascular events, including cardiovascular death, myocardial infarction, and ischemic stroke. In this study, the incidence and timing of endpoint events will be used as indicators for assessing endpoint outcomes. Researchers should keep detailed records of participants’ signs and symptoms. If the participant has any drug-related adverse events, the researchers will immediately submit it to the Ethics Review Committee of Hospital of Chengdu University of Traditional Chinese Medicine in the form of a written case report for identification.

### 3.5 Statistical analysis

The data of the study will be analyzed by statistical analysis software packages SPSS 23.0 and will be performed by experts from the Statistics Teaching and Research Office of Chengdu University of Traditional Chinese Medicine. The measurement results was calculated by *t* test, and the results were expressed as “mean ± standard deviation.” Chi-squared test was used to analyze the count data. The paired design *t* test was used for comparison the differences between before and after treatment with the same group, and the independent sample *t* test was used for comparison the differences between groups. The comparison of incidence of adverse events between the 2 groups using Chi-squared test or Fisher exact probability method. If *P* < .05, it was considered as a statistically significant difference.

### 3.6 Data management

All details about patients are recorded in the case report forms by trained and qualified investigators. After the case report form is completed, it cannot be changed arbitrarily. If correction is needed, the original record must be saved. To ensure the accuracy of the results, data entry and management were checked separately by 2 clinical observers under the guidance of statistical experts. To confirm the authenticity, the established database will be locked by researchers and statisticians until submitted to experts for statistical analysis.

## 4 Discussion

The DF is a serious public health problem in global health, which not only affects the quality of life of patients and families, but also greatly increases the financial burden on health care. DFI is an important reason for aggravating the long-term nonhealing of DF.^[31]^ The current mainstream treatment of DFI is mainly to choose sensitive antibiotics, but long-term use will make the body less sensitive or accelerate the production of drug-resistant bacteria, so more complementary therapies need to be sought. Herbal medicine foot bath has a long history of treating surgical diseases and shows the unique advantages that are different from WM, namely significant clinical efficacy and few adverse reactions. FBD is a TCM compound that has been used in DF for a long time in our hospital's endocrinology department. Due to lack of evidence on the efficacy and safety of FBD for the treatment of DFI, we need conduct a randomized, double-blind clinical trial to determine the risks and benefits of using a FBD for DFI.

As we all know, this is the 1st clinical study to treat DFI with TCM FBD. Throughout the treatment period, we closely focused on the distribution of bacterial wounds, indicators of inflammation, ulcer healing, limb function, and drug safety. We will verify that FBD can inhibit the bacterial growth of wounds through objective indicators of bacterial culture, IL-6, and TNF-α. At the same time, the overall effect of the foot bath prescription is reflected by the change in the area of the wound, the walking distance and speed, the inner diameter of the lower extremity blood vessels, the blood flow velocity, and the TCM syndrome score table. Finally, the safety evaluation was reflected by blood cell analysis, urine analysis, stool testing, liver and kidney function indicators, and so on.

However, there were plenty of limitations in our trial. On the one hand, due to the small sample size of the subjects, large-scale data are lacking to confirm the efficacy of the foot bath prescription. On the other hand, the mechanism of action of FBD is not complete, and further research is necessary. We need to observe a large sample size in future research to understand its accuracy and rationality.

## Acknowledgments

The authors are grateful to the Science and Technology Department of Sichuan Province for sponsoring this research. In addition, they also thank their partner hospitals: Mianyang Traditional Chinese Medicine Hospital and Shuangliu District Traditional Chinese Medicine Hospital.

## Author contributions

**Conceptualization:** Yuan Zhang, Haipo Yuan.

**Data curation:** Jian Kang, Hongyan Xie.

**Investigation:** Xinhua Long, Luguang Qi.

**Methodology:** Yuan Zhang, Haipo Yuan.

**Project administration:** Chunguang Xie.

**Resources:** Yuan Zhang, Jian Kang.

**Supervision:** Guangming Gong.

**Writing original draft:** Yuan Zhang, Haipo Yuan.

**Writing – review & editing:** Guangming Gong.
